# Dynamics of soil organic carbon mineralization in tea plantations converted from farmland at Western Sichuan, China

**DOI:** 10.1371/journal.pone.0185271

**Published:** 2017-09-20

**Authors:** Renhuan Zhu, Zicheng Zheng, Tingxuan Li, Xizhou Zhang, Shuqin He, Yongdong Wang, Tao Liu, Wei Li

**Affiliations:** 1 College of Resources Science, Sichuan Agricultural University, Chengdu, China; 2 College of Forestry, Sichuan Agricultural University, Chengdu, China; RMIT University, AUSTRALIA

## Abstract

Climate warming and land use change are some of the drivers affecting soil organic carbon (SOC) dynamics. The Grain for Green Project, local natural resources, and geographical conditions have resulted in farmland conversion into tea plantations in the hilly region of Western Sichuan. However, the effect of such land conversion on SOC mineralization remains unknown. In order to understand the temperature sensitivity of SOC decomposition in tea plantations converted from farmland, this study considered the different years (i.e., 2–3, 9–10, and 16–17 years) of tea plantations converted from farmland as the study site, and soil was incubated for 28 days at 15°C, 25°C, and 35°C to measure the soil respiration rate, amount, and temperature coefficient (Q_10_). Temperature and land use type interactively affected the SOC mineralization rate, and the cumulative amount of SOC mineralization in all the plots was the largest at 35°C. SOC mineralization was greater and more sensitive to temperature changes in the farmland than in the tea plantations. Compared with the control, tea plantation soils showed lower SOC mineralization rate and cumulative mineralization amount. The 16–17-year-old tea plantation with a low SOC mineralization amount and high SOC content revealed the benefits of carbon sequestration enhancement obtained by converting farmland into tea plantations. The first-order kinetic equation described SOC mineralization dynamics well. Farmland conversion into tea plantations appeared to reduce the potentially mineralizable carbon pool, and the age of tea plantations also had an effect on the SOC mineralization and sequestration. The relatively weak SOC mineralization temperature sensitivity of the tea plantation soils suggested that the SOC pool of the tea plantation soils was less vulnerable to warming than that of the control soils.

## Introduction

Soils compose the largest carbon pool in the terrestrial ecosystem [1]. Soil organic carbon (SOC) mineralization, which directly reflects the SOC decomposition, is one of the most important processes in the ecosystem carbon cycle [2]. Small changes in SOC storage significantly influence atmospheric CO_2_ concentrations and the global carbon cycle [3]. Therefore, accurate evaluation of SOC mineralization dynamics and its influencing factors has become a hotspot in the study of the terrestrial ecosystem carbon cycle.

Climate warming and land use change are some of the drivers affecting SOC mineralization [4–6]. According to related research, the SOC pool presents different responses to various temperatures, but the dynamics of SOC mineralization still remain controversial [7–8]. The temperature coefficient (Q_10_) represents the temperature sensitivity of SOC mineralization [9–11]. However, the factors that influence Q_10_ are complicated, and cause considerable spatial–temporal changes [12]. Hence, further research is necessary. Land use change is identified as a cause of SOC losses through erosion and vegetation conversion, and has become a factor contributing to the increase in atmospheric CO_2_ concentration [13]. The characteristics of SOC mineralization varied among different land use types, because the land use change could alter litterfall amount, litter chemistry and soil chemical and physical properties [14]. Many scholars have conducted extensive research on SOC mineralization and its temperature sensitivity [15–16]. However, studies on SOC mineralization in the tea plantation ecosystem are scarce.

The Grain for Green Project was implemented in the upper reaches of the Yangtze River in the 1990s. It is the largest land retirement/afforestation program in China. It was launched in 1999 to mitigate land degradation (soil erosion) by returning cultivated land to forestland or grassland. Since its implementation, the study area has presented established patterns of farmland conversion into tea plantations according to its own geographical conditions and natural resource characteristics. Tea (*Camellia sinensis* L.) is a perennial evergreen crop with root absorption characteristics and exudates. Tea plantations are artificially managed and gradually form a unique regional ecosystem [13]. Our previous studies showed that soil properties change with the increasing age of tea plantation, including the content of SOC and nutrient elements; these changed soil properties modify the soil structure and function [17–19]. However, the effect of long-term tea plantations on SOC mineralization in the hilly region of Western Sichuan, China, is unclear. This region is undergoing farmland conversion into tea plantations, and the ecological effect of such a conversion has been increasingly highlighted. Therefore, characterizations of SOC mineralization during this change in land use pattern need to be performed.

Accordingly, our main objectives of this paper were to (1) evaluate the rates of SOC mineralization in soils of tea plantations, (2) assess and quantify the short- and long-term effects of farmland conversion into tea plantations on SOC mineralization dynamics and (3) clarify the response of SOC mineralization to the effect of temperature with the conversion of farmland into tea plantations. We hypothesized that the dynamics of SOC mineralization differed across different tea plantations ages and soil depths due to the Grain for Green Project, and planting tea was beneficial for SOC sequestration in certain years. This study provides a theoretical basis for the effective conversion of farmland into tea plantations and the sustainable development of tea plantations.

## Materials and methods

### Ethics statement

On behalf of, and having obtained permission from all the authors, I declare that: the paper is not currently being considered for publication elsewhere; all authors have been personally and actively involved in substantive work leading to the report, and will hold themselves jointly and individually responsible for its content. No specific permissions were required for these locations for soil sampling, and the field studies did not involve endangered or protected species.

### Site description

**Fig 1:**
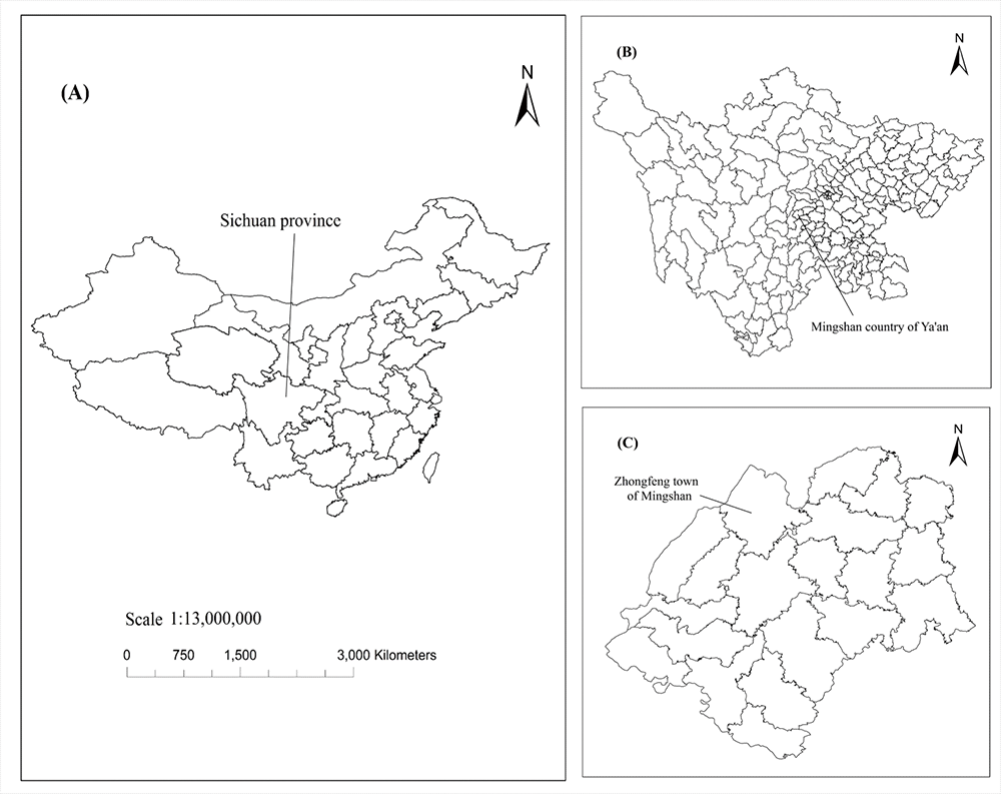
Location of the study area.

The study area is an ecological tea plantation in Zhongfeng, Mingshan District, Ya’an City (103°11′42″–103°12′02″E, 30°12′04″–30°12′43″N) (Fig 1), belong to long-term agricultural research site of Sichuan Agricultural University. This region is typical of Sichuan’s hilly areas (mean altitude of 700 m). It has a subtropical monsoon climate with a mean annual temperature of 15.4°C and a mean annual rainfall of approximately 1500 mm; 72.6% of precipitation occurs between July and September. The exposed layer is composed of sedimentary rocks mainly formed after the Mesozoic age. The yellow soil was formed in the older alluvium. Since the 1990s, the study area has become a demonstration zone of "Grain for Green Project" in the upper reaches of the Yangtze River. Based on the geographical conditions and natural resources characteristics, combined with economic development, population growth and other national policies, a new planting pattern of returning farmland to tea plantation had been formed gradually in the study area. However, some farmland and artificial woodland were still sporadically distributed. Driven by economic interests, the planting areas were constantly increasing for returning farmland to tea plantation. According to the long-term farming habits of local farmers, as well as the unified management of Sichuan Agricultural University, the agricultural management in this region had not changed significantly, so the SOC concentrations in farmland soil were considered to be essentially unchanged as well.

Tea cultivation and fertilization in the study area proceed as follows: the cultivation density of tea is set to about 8 × 10^4^ plants per ha (broad row 150 ± 15 cm; narrow row 35 ± 15 cm; the distance between two plants is 16 ± 4 cm). Swine manure (15,000 kg hm^-2^) and K_2_SO_4_ fertilizer [m(N): m(P_2_O_5_): m(K_2_O) = 20:8:8, 750 kg hm^-2^] as the base fertilizer are spread along vertical edges beneath the tree canopy in mid-October, followed by the addition of complex chemical fertilizer, swine manure, and mulch covering. A top dressing of the tea plantations is added three times a year, and the position of the top dressing is similar to that of the basal dressing. The following year in mid-February, 1500 kg hm^-2^ of complex fertilizer and 600 kg hm^-2^ of urea are applied. Complex fertilizer (750 kg hm^-2^) and urea (300 kg hm^−2^) are added again to the soils in late May and July. Tea plantations were irrigated by conventional management method [20–21].

### Soil sampling and analysis

In this study, soil samples were collected in early October 2012. Three tea plantations with different ages were selected for the field investigation. These ages included 2–3, 9–10, and 16–17 years (RT2–3, RT9–10, and RT16–17, respectively), and an unconverted farmland was used as the control (CK). All these sites were located on the same physiographical units with the same slope aspects, soil parent material, and fertilizer addition regime. The sampling site description is shown in Table 1. Five quadrants were selected from each site. Each quadrant measured 15 m × 15 m and had five sampling plots. After removing Surface litter and aboveground vegetation, three large (1~2 kg) soil bulk samples were taken with a small shovel from each sampling plot at depths of 0–10, 10–20, and 20–40 cm. Then, an S-shaped sampling method was used to mix the five soil samples of each depth as a repeat and obtain sampling points. The specific tea plantation sampling points were vertically located below the tea canopy edge. The soil samples were sealed in plastic bags and transported to the laboratory, where they were air-dried at room temperature for a week. Another separate soil sample was collected with a 100-cm^3^ cylindrical core for soil bulk density of each plot at depths of 0–10, 10–20, and 20–40 cm. The soils’ physical and chemical properties are listed in Table 2.

**Table 1:** Description of the sampling plots.

Code	Sites	Years under tea plantation	Slope degree (°)	Altitude (m)	Area (hm^2^)	Undergrowth vegetation
CK^a^	Farmland	0	28	740	≈0.20	Sparse *Setaria palmifolia* Stapf
RT2–3^b^	Tea plantation	2–3	30	742	≈0.41	Tea
RT9–10 ^c^	Tea plantation	9–10	30	742	≈0.75	Tea, *Stenoloma chusanum* Ching
RT16–17 ^d^	Tea plantation	16–17	30	742	≈0.67	Tea, Chinese fir

^a^ CK farmland

^b^ RT2-3 Tea plantation planted for 2–3 years

^c^ RT9-10 Tea plantation planted for 9–10 years

^d^ RT16-17 Tea plantation planted for 16–17 years

**Table 2:** The physical and chemical properties of soil in each sampling plot.

Soil depth (cm)	Sites	Total nitrogen (g kg^−1^)	Organic carbon (g kg^−1^)	Total nitrogen stocks (Mg ha^−1^)	Organic carbon stocks (Mg ha^−1^)	Labile organic carbon (g kg^−1^)	Microbial biomass carbon (mg kg^−1^)	Bulk density (g cm^−3^)	pH
0–10	CK	1.20A^1^a^2^	26.34Aa	1.35Ac	29.53Bc	3.62Ca	442.11Ba	1.13	4.47
	RT2–3	0.78Da	20.49Ba	0.97Bc	25.48Cc	6.68Ba	382.11Ca	1.27	4.24
	RT9–10	0.85Ca	21.54Ba	0.97Bc	24.68Cc	6.39Ba	466.42Ba	1.15	3.97
	RT16–17	1.07Ba	27.84Aa	1.36Ac	35.52Ac	8.00Aa	570.32Aa	1.28	3.79
10–20	CK	0.98Ab	22.17Bb	2.31Bb	52.13Bb	3.56Ca	357.90Bb	1.02	4.77
	RT2–3	0.71Cb	19.80Da	1.77Db	49.00Bb	6.11Bb	339.48Bb	1.21	4.18
	RT9–10	0.82Ba	20.85Ca	1.89Cb	47.96Bb	6.14Ba	468.42Aa	1.12	3.85
	RT16–17	0.90Bb	23.43Ab	2.59Ab	67.74Ab	6.61Ab	488.21Ab	1.39	3.79
20–40	CK	0.83Ac	19.65Ac	3.58Ba	85.13Da	3.15Bb	253.68Cc	0.85	5.30
	RT2–3	0.54Cc	17.15Bb	2.73Ca	86.98Ca	5.56Ab	311.06Bb	1.22	4.29
	RT9–10	0.69Bb	17.71Bb	3.65Ba	93.52Ba	5.72Ab	349.74ABb	1.30	4.08
	RT16–17	0.85Ab	19.80Ac	5.07Aa	118.28Aa	5.86Ac	378.16Ac	1.28	4.17

^1^ Different upper case letters in each column indicate significant difference among different years under tea plantation within a soil depth at *p* < 0.05 level according to the LSD test.

^2^ Different lower case letters in each column indicate significant difference among different soil depths at *p* < 0.05 level according to the LSD test.

The undisturbed soil samples were oven-dried at 105°C to a constant mass and weighted to determine the soil bulk density by dry weight. The air-dried and sieved (2 mm) soil samples were used for pH determination. Soil pH was determined with a glass electrode at a soil–water ratio of 1:2.5 [22]. The air-dried soil samples sieved to < 2 mm were ground to pass through a 0.15mm (100 mesh) plastic sieve for the determination of SOC, total N, and labile organic carbon concentrations. The potassium dichromate oxidation method of Lu [22] was applied to analyze SOC. Soil total N (TN) was determined with the semi-micro Kjeldahl method [22]. The contents of labile organic carbon were determined by oxidation using a potassium dichromate solution in sulfuric acid (1/6K_2_Cr_2_O_7_-1:3H_2_SO_4_) as described in Liu et al [23]. Half a gram of the soil sample was placed in a test tube, and 10 mL of 0.2 M mixed liquor (1/6K_2_Cr_2_O_7_-1:3H_2_SO_4_) was added to the test tube. Afterward, the test tube was boiled at 130°C–140°C for 5 min. While the test tube was cooling down, all of the solution was transferred into a 250 mL Erlenmeyer flask. The remaining K_2_Cr_2_O_7_ was titrated with 0.2 M FeSO_4_. The chloroform fumigation extraction method was used to determine the soil microbial biomass carbon [24].

### Incubation experiment

Twenty grams (equivalent dry mass) of sieved (2 mm) bulk soil was adjusted by distilled water to meet the 60% water holding capacity generally considered to be the optimum moisture content for microbial respiration [25]. Each temperature treatment and soil depth had five replicates, which were placed in 250 mL plastic bottles, and hung with a 25 mL plastic bottle containing 5 mL of 0.2 M NaOH as a CO_2_ trap. The larger plastic bottle with its matching lid was covered and incubated at 15°C, 25°C, and 35°C. The evolved CO_2_ was trapped in the 0.2 M NaOH. After precipitation, 1 mL of 0.1 M BaCl_2_ was added. The CO_2_ concentration was then determined by titration with 0.2 M HCl. Trapping and determination were conducted at increasing time intervals on days 1, 4, 7, 14, 21, and 28.

### Calculation and statistical analysis

To describe SOC mineralization under different culture temperatures, the first order kinetic equation was used as follows [26]:

$$C_{t}=C_{0}(1-e^{-kt})$$ (1)

where *C*_*t*_ the cumulative value of mineralized SOC (mg kg^-1^) during t (days), *C*_0_ is the potentially mineralizable SOC (i.e., basal carbon mineralization rate at 0°C), *t* is the incubation time, and *k* is a mineralization rate constant.

The temperature coefficient (i.e., Q_10_) is a widely used index of temperature dependence describing the proportional rate change given a 10°C temperature change [27]. Eq (2) was used to describe the temperature dependence of SOC mineralization:

$$Q_{10}=\frac{R_{\left(\text{t},T+10\right)}}{R_{\left(\text{t},T\right)}}$$ (2)

where *R* (mg kg^−1^ d^−1^) is the SOC mineralization rate, and *T* is the incubation temperature (°C).

Analysis of variance (ANOVA) was performed with the SPSS software (11.0). Two-way ANOVA, followed by the least significant difference (LSD) test (*p*<0.05), was used to compare the plots representing different tea plantation ages and incubation temperatures.

## Results

### SOC mineralization dynamics

**Fig 2:**
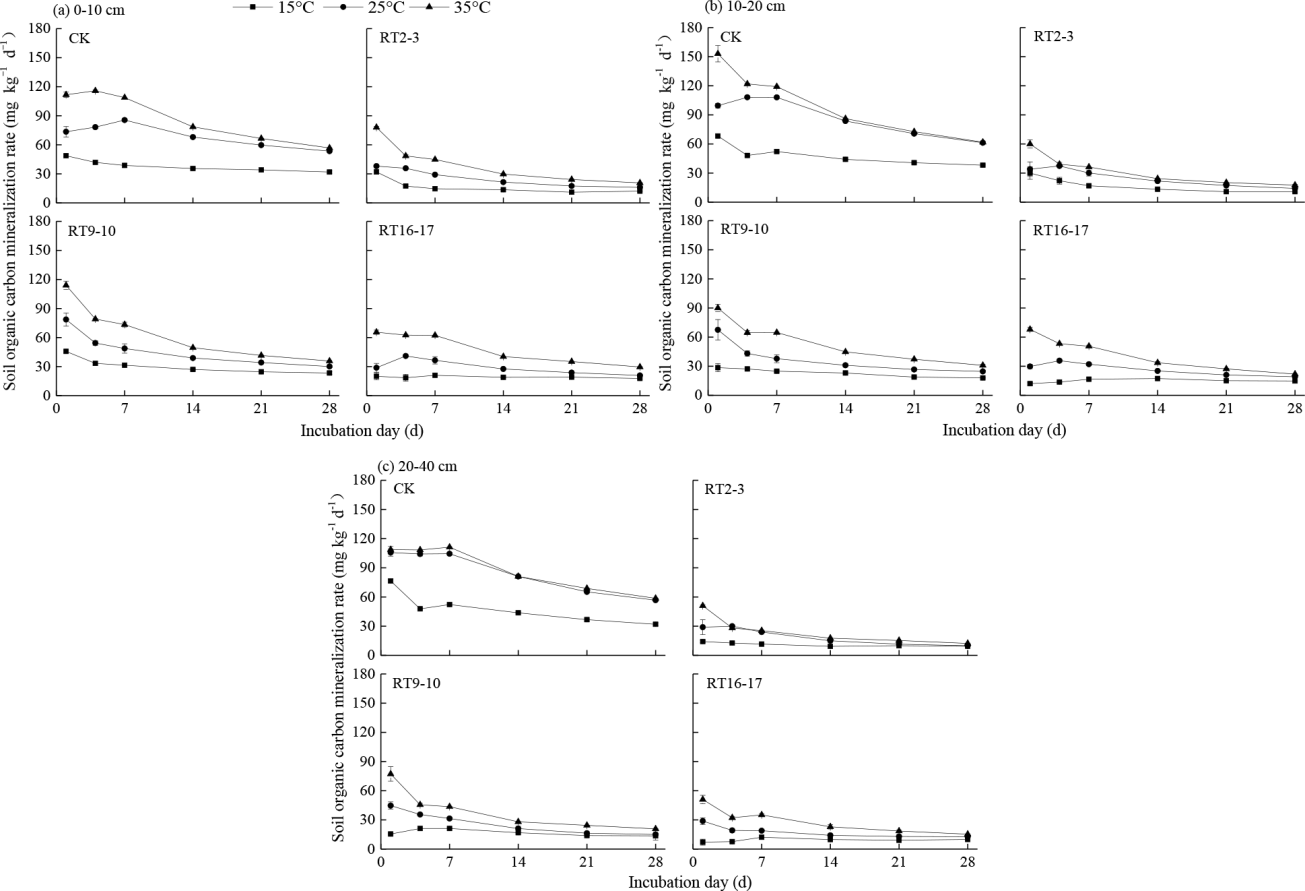
Dynamics of SOC mineralization rate of farmland conversion to tea plantations at three soil layers.

The daily and cumulative SOC mineralization dynamics during the 28-day incubation are shown in Fig 2. The daily SOC mineralization amount at each soil depth was in the order of 35°C > 25°C > 15°C at all the plots. Obvious changes occurred under 25°C and 35°C. Under the three different incubation temperatures, the daily and cumulative SOC mineralization displayed similar trends in the three depths at four plots in the order of CK > RT9–10 > RT16–17 > RT2–3.

The mineralized SOC rate reached the maximum value on the first day of incubation and significantly decreased and stabilized after 21 d for the 0–10 cm depth soil (Fig 2A). However, different decreasing amplitudes of the mineralization rate were observed among the three tea plantations. The decrease was fast in RT9–10 and RT2–3 and slow in RT16–17, indicating that the mineralization rate did not increase as the SOC content increased. The change in the daily mineralization amount of the 10–20 cm soil layers was similar to that in the surface soils at 0–10 cm depth (Fig 2B). However, the SOC mineralization rate at all plots at 20–40 cm depth changed slightly under the three temperatures during the incubation (Fig 2C).

**Fig 3:**
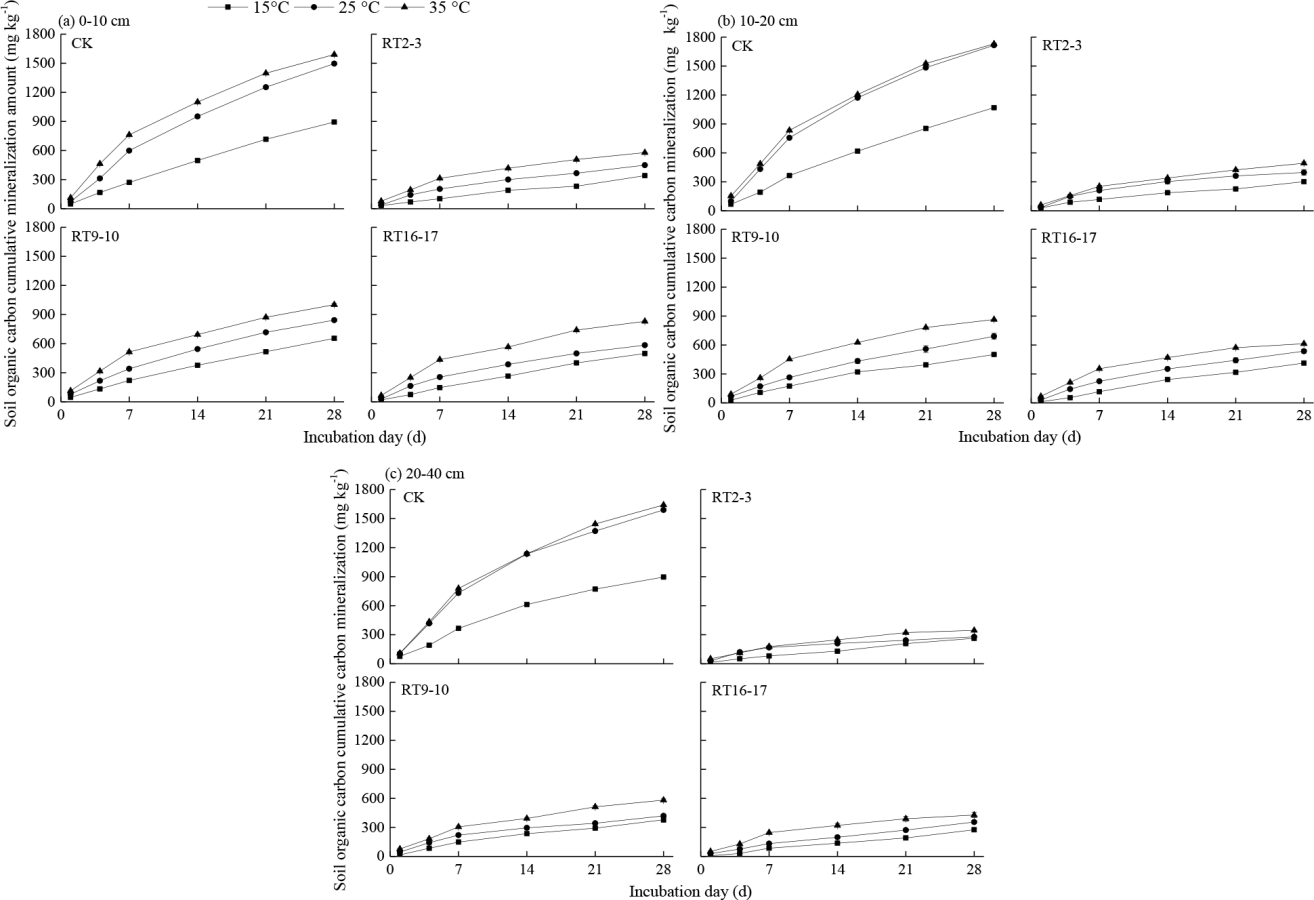
The accumulation amount of SOC mineralization of farmland conversion to tea plantations at three soil layers.

The changes in the accumulation amount of SOC mineralization were similar to the changes in mineralization rate (Fig 3A–3C). The total mineralized carbon ranged from 263.25–1068.75 mg kg^−1^ under 15°C, from 278.25–1715.25 mg kg^−1^ under 25°C, and from 345.00–1730.00 mg kg^−1^ under 35°C during the 28-day incubation (Table 3). The amounts of total mineralized carbon significantly increased with temperature at all the plots, but the range of increase decreased. The results showed that the SOC cumulative mineralization amount significantly increased with temperature at all the plots. The total mineralized carbon of RT9–10 was significantly higher than that of RT16–17 and RT2–3. The accumulation amounts of SOC mineralization in the tea plantations were significantly lower than that of the control. The trend of the change in mineralized percentage was similar to that of the mineralization cumulative amount.

**Table 3:** SOC mineralization characteristics during the course of farmland conversion to tea plantations.

Soil depth (cm)	Sites	Cumulative mineralization amount (mg kg^−1^)			Mineralized percentage		
		15°C	25°C	35°C	15°C	25°C	35°C
0–10	CK	897.75C^1^a^2^	1496.00Ba	1589.75Aa	3.41Ca	5.68Ba	6.03Aa
	RT2–3	340.50Cd	449.00Bd	578.50Ad	1.66Cc	2.19Bc	2.82Ab
	RT9–10	656.25Cb	846.00Bb	1001.50Ab	3.05Cb	3.93Bb	4.65Aa
	RT16–17	498.00Cc	585.00Bc	829.50Ac	1.79Cc	2.10Bd	2.98Ab
10–20	CK	1068.75Ca	1715.25Ba	1730.00Aa	4.82Ca	7.74Ba	7.80Aa
	RT2–3	299.25Cd	396.75Bd	492.75Ac	1.51Cd	2.00Bd	2.49Ac
	RT9–10	500.25Cb	688.50Bb	864.50Ab	2.40Cb	3.30Bb	4.15Ab
	RT16–17	412.50Bc	533.25Ac	613.25Ad	1.76Bc	2.28Ac	2.62Ad
20–40	CK	897.00Ca	1588.75Ba	1641.50Aa	4.57Ca	8.09Ba	8.36Aa
	RT2–3	263.25Bc	278.25Bd	345.00Ac	1.54Bc	1.62Bd	2.01Ac
	RT9–10	379.50Bb	420.00Bb	582.00Ab	2.15Bb	2.37Bb	3.29Ab
	RT16–17	276.00Cc	354.00Bc	428.25Ad	1.40Cc	1.79Bc	2.16Ad

^1^ Different upper case letters in each row indicate significant difference among different culture temperatures at *p* < 0.05 level according to the LSD test.

^2^ Different lower case letters in each column indicate significant difference among different years under tea plantation within a soil depth at *p* < 0.05 level according to the LSD test.

### SOC mineralization temperature sensitivity

The calculated Q_10_ values ranged from 1.15–1.40 and varied with soil depths for all plots (Table 4). The Q_10_ value of the 15°C–25°C interval was higher than that of the 25°C–35°C interval. Meanwhile, the temperature sensitivities of SOC mineralization of farmland and tea plantations differed significantly, and the Q_10_ value of tea plantations was significantly lower than that of the control. In addition, except for RT2–3, the Q_10_ value at 20–40 cm soil depth was not less than that at the 0–10 and 10–20 cm depths.

**Table 4:** Kinetic parameters for SOC mineralization.

Soil depth (cm)	Sites	*C*_0_			k			R^2^			Q_10_
		15°C	25°C	35°C	15°C	25°C	35°C	15°C	25°C	35°C	
0–10	CK	2000.02	2104.84	1981.74	0.021	0.044	0.058	1.000	0.997	1.000	1.37
	RT2–3	520.17	536.53	597.99	0.038	0.063	0.097	0.994	0.993	0.985	1.30
	RT9–10	1055.82	1082.44	1128.40	0.035	0.052	0.073	0.999	0.996	0.992	1.24
	RT16–17	1110.60	842.93	909.51	0.021	0.042	0.080	1.000	1.000	0.986	1.30
10–20	CK	1977.37	2095.29	2108.09	0.027	0.060	0.061	0.998	0.998	0.999	1.31
	RT2–3	370.48	415.75	522.80	0.053	0.100	0.085	0.969	0.996	0.984	1.29
	RT9–10	752.05	925.88	941.98	0.038	0.047	0.085	0.996	0.993	0.994	1.32
	RT16–17	566.44	749.45	652.86	0.039	0.056	0.100	0.996	0.995	1.000	1.22
20–40	CK	1171.32	1850.26	1918.36	0.052	0.068	0.067	0.997	0.998	0.996	1.40
	RT2–3	208.49	269.59	374.82	0.070	0.131	0.088	1.000	0.976	0.986	1.15
	RT9–10	534.27	624.26	666.42	0.042	0.039	0.070	0.990	0.987	0.985	1.25
	RT16–17	324.41	588.00	483.51	0.042	0.044	0.078	0.995	0.998	1.000	1.25

### SOC mineralization dynamic simulation

SOC mineralization at all depths responded to temperature following a first-order kinetic equation at all the sites (Table 4). First, the minimum coefficient of determination (R^2^) value for all the fitting equations was 0.976, indicating that the equation effectively described the dynamic process of SOC mineralization. Second, the potentially mineralizable carbon (*C*_0_) generally increased with temperature and was consistent with the trend of cumulative mineralization amount. Third, the *C*_0_ of farmland converted into tea plantations was lower than that of the control and presented a similar trend as the total mineralized carbon arranged in a descending order (i.e., RT9–10 > RT16–17 > RT2–3).

## Discussions

### SOC mineralization rate and amount with farmland conversion into tea plantations

SOC mineralization rates were high during the first four days of incubation and became low thereafter, consistent with some studies of agricultural [28] and forested [14] soils. The highest daily SOC mineralization was obtained on the first day of incubation possibly because of the activating effects of microorganisms following the short-term dried soil rewetting [29]. The SOC mineralization rates decreased with the extension of incubation time [30–31], due to the decrease of labile organic carbon, which is easily degraded by microorganisms. [32].

SOC mineralization is a complex biogeochemical process that is affected by many direct and indirect factors, such as temperature, water content and carbon input [5, 32]. Accordingly, temperature is much more important than the other factors in terms of affecting the mineralization process of organic matter [33]. The influence of temperature on SOC mineralization varies for different temperature ranges [33] and the SOC mineralization rate generally increase significantly with the increase of temperature [16]. In this study, the SOC mineralization rate and amount presented a similar trend (i.e., both were significantly higher at 35°C), which is in line with the results of previous studies [31–32]. It is found that stimulated warming can enhance CO_2_ efflux in both laboratory incubation experiments and field [14, 16], due to the increased microbial activity and enhanced soil respiration [4]. However, the current study showed that the increasing degree of total mineralized carbon in almost all the plots decreased with temperature possibly because of the acclimatization of soil respiration to warming [34].

Given the combined effects of various biotic and abiotic factors and the differences in human disturbance, the mineralization process and mineralization rate of SOC differ under different land use patterns [35–36]. In this study, the SOC mineralization rates of tea plantations were lower compared with that of the control, indicating that the conversion of farmland into tea plantation was beneficial to the enhancement of SOC stability This condition may be attributed to the differences in the stability of soil structure and the amount and nature of litter input [6,32]. In the initial conversion stage (RT2-3), the amount of litter was small, and rapid decreases in SOC were likely caused by the destruction of soil aggregates by human activities (e. g., regular picking of tea, weeding, tillage and fertilization) in tea gardens [21]. Soil microorganisms drive cycling and storage of soil C through decomposition of plant root and litter inputs [37]. Soil microbial biomass carbon (MBC), which is constituted by archaea, bacteria and eukaryotes, has been correlated with microbial diversity and enzyme activities in the soil [38]. It was not conductive to the breeding of microorganisms and the development of biodiversity, in the case of a decrease in the aboveground litter inputs and an increase in anthropogenic disturbance in the initial conversion stage [39]. Thus, the mineralization rate in RT2-3 was lower compared with control. During the 9–10 years of tea plantation, the mineralizable carbon increased because the amount of litter increased and was returned to the soil [18]. Plant residues and metabolism secretions accumulated continuously with the increase of tea planting years, resulting in the recovery of microbial numbers and activity to some extent, which led to the rebound of SOC. In addition, tea plant litter contains substances (e.g., polyphenol, lignin, and tannin) that are difficult to decompose, retarding the mineralization of SOC [40–41]. The SOC content in RT16–17 was significantly higher than that in CK, but the total mineralized carbon was lower, indicating that the tea plantation of 16–17 years resulted in increased soil carbon sink. The control had a relatively higher degree of organic residue and SOC decomposition. This condition may be attributed to an increase in the carbon–nitrogen ratio of RT16–17, and the decreased nitrogen content limited SOC mineralization to some extent [16]. Additionally, lower pH could decrease microbial growth [4], and thus limit microbial decomposition and lead to greater SOC sequestration in RT16-17.

Alvarez and Alvarez [42] applied exponential and hyperbolic models to describe SOC mineralization during incubation. Their results showed that the exponential model was more capable of describing SOC mineralization patterns than the hyperbolic model in a wide range of soil management practices and depths. In the current study, we fitted SOC mineralization dynamics by using the same exponential equation (Table 4). This equation effectively described the mineralization process. According to the change in C_0_, farmland conversion into tea plantations effectively enhanced carbon sequestration. In addition, C_0_ decreased in RT16–17 exhibited a carbon sink effect, suggesting that long-term tea planting was beneficial to SOC sequestration.

### SOC mineralization temperature sensitivity with farmland conversion into tea plantations

The Q_10_ value is usually used to predict the response of SOC mineralization and sequestration to future warming [43]. Several studies have demonstrated that the temperature sensitivity of SOC mineralization changes with warming temperature [5,44–45]. In this study, the Q_10_ value decreased with the increase in temperature, which is consistent with the results of the majority of previous studies [46–47]. The high Q_10_ value of the 15°C–25°C interval suggests that thermal adaptation affected soil heterotrophic respiration. The different responses to temperatures might be due to the changes in the microbial community structures and the incubated physiochemical properties [4, 14].

The Q_10_ value of tea plantations was significantly low because, first, the nitrogen content of the control was higher than that of the tea plantations, and the C/N ratio of the control was lower. The nitrogen affected the microbial activity in a complex manner [48]. A low C/N ratio increases the carbon mineralization rate, and a high C/N ratio imposes certain restrictions on soil microbial activities. Thus, organic carbon decomposition and mineralization slowed down, allowing an increase in SOC fixation ability. Second, the Q_10_ values were affected by vegetation type [31], substrate availability [49], and soil water content [50]. The conversion of land use changed soil porosity and permeability, which influenced SOC mineralization temperature sensitivity [51]. The relatively weak SOC mineralization temperature sensitivity of the tea plantation soils suggested that the SOC pool of the tea plantation soils was less vulnerable to warming than that of the control soils with a large Q_10_ value. In addition, some studies showed that different SOC fractions had remarkably different Q_10_ values [52]. Tea plantations with different conversion years had different SOC mineralization temperature sensitivity, and this might be due to the changes of SOC composition [19].

Generally, SOC at different depths had different responses to changes in soil temperature, owing to the difference in SOC stability and microbial selectivity to C utilization [53]. Research has pointed out that deeper soil layers are less temperature-sensitive than upper soil layers [44], and suggested that the change of Q_10_ with depths was related to substrate availability [16]. However, the Q_10_ value for the 20–40 cm depth in this study was not lower than that of the 0–10 and 10–20 cm depths, indicating that the deeper soil layer of the study area was as sensitive as the topsoil to warming. This result was consistent with some previous studies in which the Q_10_ of SOC mineralization in the subsoil was higher than the topsoil, due to the larger proportion of recalcitrant and lower quality organic fractions [16, 54]. Hence, further attention should be paid to deeper soil layers of tea plantation.

## Conclusions

The cumulative amount of SOC mineralization was significantly high at 35°C in all the plots, but the increasing amplitude of the 25°C–35°C interval was less than that of the 15°C–25°C interval. SOC mineralization was greater and more sensitive to temperature changes in the farmland than in the tea plantations. The significantly low cumulative mineralization amount and Q_10_ value of tea plantations showed that farmland conversion into tea plantations effectively enhanced the soil carbon sink in the study area, especially in tea plantations aged 16–17 years, which had a low mineralization rate and a high SOC content. According to the Q_10_ value at 20–40 cm depth, the contribution of deeper layers to SOC mineralization should not be ignored. The first-order kinetic equation effectively described the SOC mineralization dynamics in the study area. Therefore, farmland conversion into tea plantations effectively reduced the potentially mineralizable carbon pool and was conducive for activated carbon accumulation.

## References

[1] van Wesemael B, Paustian K, Andrén O, Cerri CE, Dodd M, Etchevers J, et al How can soil monitoring networks be used to improve predictions of organic carbon pool dynamics and CO_2_ fluxes in agricultural soils? Plant and Soil. 2011; 338(1–2): 247–59.

[2] Bahn M, Rodeghiero M, Anderson-Dunn M, Dore S, Gimeno C, Drösler M, et al Soil respiration in European grasslands in relation to climate and assimilate supply. Ecosystems. 2008; 11(8): 1352–67. doi: 10.1007/s10021-008-9198-0 20936099 10.1007/s10021-008-9198-0 PMC2950939

[3] Wang W, Zeng W, Chen W, Zeng H, Fang J. Soil respiration and organic carbon dynamics with grassland conversions to woodlands in temperate China. PloS One. 2013; 8(8): e71986 doi: 10.1371/journal.pone.0071986 24058408 10.1371/journal.pone.0071986 PMC3751950

[4] Lu S, Zhang Y, Chen C, Xu Z, Guo X. Plant–soil interaction affects the mineralization of soil organic carbon: evidence from 73-year-old plantations with three coniferous tree species in subtropical Australia. Journal of Soils and Sediments. 2017; 17(4): 985–95.

[5] Sierra CA, Trumbore SE, Davidson EA, Vicca S, Janssens I. Sensitivity of decomposition rates of soil organic matter with respect to simultaneous changes in temperature and moisture. Journal of Advances in Modeling Earth Systems. 2015; 7(1): 335–56.

[6] Wang Q, Zeng Z, Zhong M. Soil Moisture Alters the Response of Soil Organic Carbon Mineralization to Litter Addition. Ecosystems. 2016; 19(3): 450–60.

[7] Taylor PG, Cleveland CC, Wieder WR, Sullivan BW, Doughty CE, Dobrowski SZ, et al Temperature and rainfall interact to control carbon cycling in tropical forests. Ecology Letters. 2017; 20: 779–88. doi: 10.1111/ele.12765 28414883 10.1111/ele.12765

[8] Muñoz-Rojas M, Doro L, Ledda L, Francaviglia R. Application of CarboSOIL model to predict the effects of climate change on soil organic carbon stocks in agro-silvo-pastoral Mediterranean management systems. Agriculture, Ecosystems and Environment. 2015; 202: 8–16.

[9] Craine JM, Fierer N, McLauchlan KK, Elmore AJ. Reduction of the temperature sensitivity of soil organic matter decomposition with sustained temperature increase. Biogeochemistry. 2013; 113(1–3): 359–68.

[10] Fang Y, Singh BP, Singh B. Temperature sensitivity of biochar and native carbon mineralisation in biochar-amended soils. Agriculture, Ecosystems and Environment. 2014; 191: 158–67.

[11] Sun J, He F, Zhang Z, Shao H, Xu G. Temperature and moisture responses to carbon mineralization in the biochar-amended saline soil. Science of The Total Environment. 2016; 569: 390–4. doi: 10.1016/j.scitotenv.2016.06.082 27348703 10.1016/j.scitotenv.2016.06.082

[12] Davidson EA, Belk E, Boone RD. Soil water content and temperature as independent or confounded factors controlling soil respiration in a temperate mixed hardwood forest. Global Change Biology. 1998; 4(2): 217–27.

[13] Chen CP, Juang KW, Cheng CH, Pai CW. Effects of adjacent land-use types on the distribution of soil organic carbon stocks in the montane area of central Taiwan. Botanical Studies. 2016; 57(1): 32–9 doi: 10.1186/s40529-016-0147-5 28597442 10.1186/s40529-016-0147-5 PMC5430586

[14] Sun S, Liu J, Chang SX. Temperature sensitivity of soil carbon and nitrogen mineralization: impacts of nitrogen species and land use type. Plant and Soil. 2013; 372(1): 597–608.

[15] Grellier S, Janeau JL, Hoai ND, Kim CNT, Phuong QLT, Thu TPT, et al Changes in soil characteristics and C dynamics after mangrove clearing (Vietnam). Science of The Total Environment. 2017; 593: 654–63. doi: 10.1016/j.scitotenv.2017.03.204 28364605 10.1016/j.scitotenv.2017.03.204

[16] Tian Q, Wang X, Wang D, Wang M, Liao C, Yang X, et al Decoupled linkage between soil carbon and nitrogen mineralization among soil depths in a subtropical mixed forest. Soil Biology and Biochemistry. 2017; 109:135–44.

[17] Wang SQ, Zheng ZC, Li TX, Li Y. Effects of age of tea plantations on distribution of exchangeable base cations in soil aggregates. Acta Pedologica Sinica. 2013; 50(5): 1014–21.

[18] Li W, Zheng ZC, Li TX, Wang YD. Effects of returning farmland to tea on soil organic carbon pool of hilly region in the western Sichuan. Scientia Agricultura Sinica. 2014; 47(8): 1642–51.

[19] Wang SQ, Li TX, Zheng ZC. Effect of tea plantation age on the distribution of soil organic carbon and nutrient within micro-aggregates in the hilly region of western Sichuan, China. Ecological Engineering. 2016; 90: 113–9.

[20] Shen JG. Irrigation project and benefit in tea plantation. Transactions of CSAE. 1992; 8(1): 35–41.

[21] Yang YJ. Tea cultivation in China. Shanghai Scientific and Technical Publishers. 2005.

[22] Lu RK. Soil Analytical Methods of Agronomic Chemical. China Agricultural Science and Technology Press, Beijing 2000.

[23] Liu HM, Yang ZX, Liu SQ. Methods for determining labile orange matter in different sized soil particles of different soils. Ecology and Environment. 2008; 17(5): 2046–9.

[24] Vance ED, Brookes PC, Jenkinson DS. An extraction method for measuring soil microbial biomass C. Soil biology and Biochemistry. 1987; 19(6): 703–7.

[25] Howard DM, Howard PJA. Relationships between CO_2_ evolution, moisture content and temperature for a range of soil types. Soil Biology and Biochemistry. 1993; 25(11): 1537–46.

[26] Canali S, Trinchera A, Intrigliolo F, Pompili L, Nisini L, Mocali S, et al Effect of long term addition of composts and poultry manure on soil quality of citrus orchards in Southern Italy. Biology and Fertility of Soils. 2004; 40(3): 206–10.

[27] Kirschbaum MU. The temperature dependence of soil organic matter decomposition, and the effect of global warming on soil organic C storage. Soil Biology and biochemistry. 1995; 27(6): 753–60.

[28] Haddix ML, Plante AF, Conant RT, Six J, Steinweg JM, Magrinibair K, et al The role of soil characteristics on temperature sensitivity of soil organic matter. Soil Science Society of America Journal. 2011; 75(1): 56–68.

[29] Sparling GP, Speir TW, Whale KN. Changes in microbial biomass C, ATP content, soil phospho-monoesterase and phospho-diesterase activity following air-drying of soils. Soil biology and biochemistry. 1986; 18(4): 363–70.

[30] Yang L, Pan J, Shao Y, Chen JM, Ju WM, Shi X, et al Soil organic carbon decomposition and carbon pools in temperate and sub-tropical forests in China. Journal of environmental management. 2007; 85(3): 690–5. doi: 10.1016/j.jenvman.2006.09.011 17107746 10.1016/j.jenvman.2006.09.011

[31] Wang G, Zhou Y, Xu X, Ruan H, Wang J. Temperature sensitivity of soil organic carbon mineralization along an elevation gradient in the Wuyi Mountains, China. PloS One. 2013; 8(1): e53914 doi: 10.1371/journal.pone.0053914 23342038 10.1371/journal.pone.0053914 PMC3544745

[32] Jia J, Yu D, Zhou W, Zhou L, Bao Y, Meng Y, et al Variations of soil aggregates and soil organic carbon mineralization across forest types on the northern slope of Changbai Mountain. Acta Ecologica Sinica. 2015; 35(2): 1–7.

[33] Arevalo C, Chang SX, Bhatti JS, Sidders D. Mineralization potential and temperature sensitivity of soil organic carbon under different land uses in the parkland region of Alberta, Canada. Soil Science Society of America Journal. 2012; 76(1): 241–51.

[34] Wan S, Norby RJ, Ledford J, Weltzin JF. Responses of soil respiration to elevated CO_2_, air warming, and changing soil water availability in a model old‐field grassland. Global Change Biology. 2007; 13(11): 2411–24.

[35] Moscatelli MC, Tizio AD, Marinari S, Grego S. Microbial indicators related to soil carbon in Mediterranean land use systems. Soil and Tillage Research. 2007; 97(1): 51–9.

[36] Wu JL, Liu MY, Zhao GQ, Yu YL, Liu LW, Liu H. Effects of land-use types on soil organic carbon mineralization and greenhouse gas emissions in Loess tableland. Journal of Agro-Environment Science. 2016; 35(5): 1006–15.

[37] Quan L, Song X, Gu H, Fei G. Nitrogen deposition and management practices increase soil microbial biomass carbon but decrease diversity in Moso bamboo plantations. Scientific Reports. 2016; 6: 28235 doi: 10.1038/srep28235 27302857 10.1038/srep28235 PMC4908385

[38] Glaciela K, Odair A, Mariangela H. Three decades of soil microbial biomass studies in Brazilian ecosystems: Lessons learned about soil quality and indications for improving sustainability. Soil Biology and Biochemistry. 2010; 42: 1–13.

[39] Liu Y, Wei X, Guo X, Niu D, Zhang J, Xia G, et al The long-term effects of reforestation on soil microbial biomass carbon in sub-tropic severe red soil degradation areas. Forest Ecology and Management. 2012; 285: 77–84.

[40] Khan SH. The use of green tea (*Camellia sinensis*) as a phytogenic substance in poultry diets. Onderstepoort Journal of Veterinary Research. 2014; 81(1): 1–8. 10.4102/ojvr.v81i1.706 24833345

[41] Fan D, Fan K, Yu C, Lu Y, Wang X. Tea polyphenols dominate the short-term tea (*Camellia sinensis*) leaf litter decomposition. Journal of Zhejiang University-SCIENCE B (Biomedicine & Biotechnology). 2017; 18(1): 99–108. 10.1631/jzus.B1600044 PMC5296227 28124839

[42] Alvarez R, Alvarez CR. Soil organic matter pools and their associations with carbon mineralization kinetics. Soil Science Society of America Journal, 2000, 64(1): 184–9.

[43] Ghosh A, Bhattacharyya R, Dwivedi BS, Meena MC, Agarwal BK, Mahapatra P, et al Temperature sensitivity of soil organic carbon decomposition as affected by long-term fertilization under a soybean based cropping system in a sub-tropical Alfisol. Agriculture Ecosystems and Environment. 2016; 233: 202–13.

[44] Gillabel J, Cebrian-Lopez B, Six J, Merckx R. Experimental evidence for the attenuating effect of SOM protection on temperature sensitivity of SOM decomposition. Global Change Biology. 2010; 16(10): 2789–98.

[45] Zhang J, Loynachan TE, Raich JW. Artificial soils to assess temperature sensitivity of the decomposition of model organic compounds: effects of chemical recalcitrance and clay‐mineral composition. European Journal of Soil Science. 2011; 62(6): 863–73.

[46] Leifeld J, Fuhrer J. The temperature response of CO_2_ production from bulk soils and soil fractions is related to soil organic matter quality. Biogeochemistry. 2005; 75(3): 433–53.

[47] Qiu X, Lü MK, Huang JX, Li W, Zhao BJ, Zhang H, et al Characteristics of soil organic carbon mineralization at different temperatures in severely eroded red soil. Chinese Journal of Plant Ecology. 2016; 40(3): 236–45.

[48] Updegraff K, Bridgham SD, Pastor J, Weishampel P, Harth C. Response of CO_2_ and CH_4_ emissions from peatlands to warming and water table manipulation. Ecological Applications. 2001; 11(2): 311–26.

[49] Gershenson A, Bader NE, Cheng W. Effects of substrate availability on the temperature sensitivity of soil organic matter decomposition. Global Change Biology. 2009; 15(1): 176–83.

[50] Suseela V, Conant RT, Wallenstein MD, Dukes JS. Effects of soil moisture on the temperature sensitivity of heterotrophic respiration vary seasonally in an old—field climate change experiment. Global Change Biology. 2012; 18(1): 336–48.

[51] Tjoelker MG, Oleksyn J, Reich PB. Modelling respiration of vegetation: evidence for a general temperature‐dependent Q_10_. Global Change Biology. 2001; 7(2): 223–30.

[52] Li D, Schädel C, Haddix ML, Paul EA, Conant R, Li J, et al Differential responses of soil organic carbon fractions to warming: Results from an analysis with data assimilation. Soil Biology and Biochemistry. 2013; 67(4): 24–30.

[53] Chang S X, Shi Z, Thomas BR. Soil respiration and its temperature sensitivity in agricultural and afforested poplar plantation systems in northern Alberta. Biology and Fertility of Soils. 2016; 52(5): 629–41.

[54] Jin X, Wang S, Zhou Y. Microbial CO_2_ production from surface and subsurface soil as affected by temperature, moisture, and nitrogen fertilisation. Soil Research. 2008; 46(3): 273–80.

